# Supplementary material for: PP-GWAS: Privacy Preserving Multi-Site Genome-wide Association Studies
Source: Nat Commun. 2025 Dec 9;16:11030. doi: 10.1038/s41467-025-66771-z (PMC12695980; doi:10.1038/s41467-025-66771-z)
Supplement: Supplementary file 1 — Supplementary Information [file 41467_2025_66771_MOESM1_ESM.pdf]

PP-GWAS: Privacy Preserving Multi-Site Genome-wide Association Studies

Supplementary Note 1: Accuracy Analysis on Simulated Data

To further assess the accuracy of PP-GWAS on simulated data, we report the squared Pearson correlation coefficient (r^2) between the negative logarithm of p-values obtained from PP-GWAS and those computed by REGENIE pertaining to the experiments whose runtimes are illustrated in Figure 3 in the main manuscript. We demonstrate that PP-GWAS can reliably reproduce the results from plaintext computation under diverse settings (Supplementary Table 1). The minimal discrepancies observed are restricted to a small number of SNPs with very high $-\log_{10}(p)$ values. These deviations are attributed to floating-point arithmetic errors, and do not affect the significance ranking of top associations, as further seen in Figure 7 in the main manuscript.

Supplementary Table 1: Agreement between PP-GWAS and REGENIE on synthetic datasets varying sample size (N), number of SNPs (M), covariates (C), and computational parties (P). Across all SNPs, the correlation of $-\log_{10}$(p-values) was $r^2 = 0.999999 \sim 1.00$ (df $= M - 2$), $p < 10^{-6}$, 95% CI [0.999999, 1.000000]; all scenarios showed near-perfect agreement ($r^2 \approx 1$)[†].

N	M	C	P	r^2
9 178	612 794	10	2	0.999999
9 178	612 794	10	4	0.999999
9 178	612 794	10	6	0.999999
9 178	612 794	10	8	0.999999
9 178	1 225 588	10	2	0.999999
9 178	1 838 382	10	2	1.000000
9 178	2 451 176	10	2	1.000000
9 178	612 794	20	2	0.999999
9 178	612 794	30	2	0.999999
9 178	612 794	40	2	0.999999
18 356	612 794	10	2	0.999999
27 534	612 794	10	2	0.999999
36 712	612 794	10	2	0.999999

[†] r^2 values are rounded to six decimal places (precision 10^{-6}).
