# Supplementary material for: PP-GWAS: Privacy Preserving Multi-Site Genome-wide Association Studies
Source: Nat Commun. 2025 Dec 9;16:11030. doi: 10.1038/s41467-025-66771-z (PMC12695980; doi:10.1038/s41467-025-66771-z)
Supplement: Supplementary file 2 — Reporting Summary [file 41467_2025_66771_MOESM2_ESM.pdf]

nature portfolio

Corresponding author(s): Arjhun Swaminathan

Last updated by author(s): Nov 11, 2025

Reporting Summary

Nature Portfolio wishes to improve the reproducibility of the work that we publish. This form provides structure for consistency and transparency in reporting. For further information on Nature Portfolio policies, see our Editorial Policies and the Editorial Policy Checklist.

Statistics

For all statistical analyses, confirm that the following items are present in the figure legend, table legend, main text, or Methods section.

n/a	Confirmed	
☐	☒	The exact sample size (*n*) for each experimental group/condition, given as a discrete number and unit of measurement
☐	☒	A statement on whether measurements were taken from distinct samples or whether the same sample was measured repeatedly
☐	☒	The statistical test(s) used AND whether they are one- or two-sided<br>*Only common tests should be described solely by name; describe more complex techniques in the Methods section.*
☐	☒	A description of all covariates tested
☒	☐	A description of any assumptions or corrections, such as tests of normality and adjustment for multiple comparisons
☐	☒	A full description of the statistical parameters including central tendency (e.g. means) or other basic estimates (e.g. regression coefficient) AND variation (e.g. standard deviation) or associated estimates of uncertainty (e.g. confidence intervals)
☐	☒	For null hypothesis testing, the test statistic (e.g. *F*, *t*, *r*) with confidence intervals, effect sizes, degrees of freedom and *P* value noted<br>*Give P values as exact values whenever suitable.*
☒	☐	For Bayesian analysis, information on the choice of priors and Markov chain Monte Carlo settings
☒	☐	For hierarchical and complex designs, identification of the appropriate level for tests and full reporting of outcomes
☐	☒	Estimates of effect sizes (e.g. Cohen's *d*, Pearson's *r*), indicating how they were calculated

Our web collection on statistics for biologists contains articles on many of the points above.

Software and code

Policy information about availability of computer code

Data collection	We used pysnptools (pysnptools==0.5.10) to generate synthetic data for our experiments.
Data analysis	We used plink (PLINK 2.0) to perform meta-analysis, and REGENIE to perform association studies for comparison. PP-GWAS code is provided in https://github.com/mdppml/PP-GWAS

For manuscripts utilizing custom algorithms or software that are central to the research but not yet described in published literature, software must be made available to editors and reviewers. We strongly encourage code deposition in a community repository (e.g. GitHub). See the Nature Portfolio guidelines for submitting code & software for further information.

Data

Policy information about availability of data

All manuscripts must include a data availability statement. This statement should provide the following information, where applicable:

- Accession codes, unique identifiers, or web links for publicly available datasets
- A description of any restrictions on data availability
- For clinical datasets or third party data, please ensure that the statement adheres to our policy

The real-world datasets analysed here are available via controlled access from the NCBI database of Genotypes and Phenotypes (dbGaP). The bladder cancer risk dataset (n=13,060; phs000346.v2.p2) and the age-related macular degeneration dataset (n=22,683; phs001039.v1.p1) contain individual-level genomic and phenotypic information collected under informed consent and are therefore available only to qualified researchers under the Data Use Limitations specified in each dbGaP record. Access requests should be submitted through the dbGaP Authorized Access system, citing the accession numbers above and including an institutional

Data Use Certification and, where applicable, IRB/ethics approval. Requests are reviewed by the appropriate NIH dbGaP Data Access Committee; the authors are not involved in approval decisions. Further details on the original study protocols, including participant recruitment and sample collection, are provided in the respective dbGaP records. Access requests are typically reviewed by the NIH Data Access Committee in about two weeks on average; if approved, dataset access is granted for one year and may be renewed. Synthetic data were generated using pysnptools. Instructions and scripts for generating these synthetic datasets are publicly available in our GitHub repository. No other custom datasets were generated for this study. Source data are provided with this paper for all figures and tables derived from testing on the synthetic data.

Research involving human participants, their data, or biological material

Policy information about studies with human participants or human data. See also policy information about sex, gender (identity/presentation), and sexual orientation and race, ethnicity and racism.

Reporting on sex and gender	This study did not involve sex- or gender-based analyses.
Reporting on race, ethnicity, or other socially relevant groupings	Race or ethnicity data were not used in our analyses.
Population characteristics	We evaluation our method on both synthetic data generated using pysnptools and two real-world datasets obtained from dbGaP: a bladder cancer dataset (n=13,060; accession number phs000346.v2.p2) and an age-related macular degeneration (AMD) dataset (n=22,683; accession number phs001039.v1.p1). All relevant information is documented in the respective dbGaP records. All data were provided in de-identified form.
Recruitment	No new participants were recruited for this study.
Ethics oversight	All human data were previously collected under appropriate Institutional Review Board (IRB) oversight, and we accessed them via dbGaP under an approved data-use agreement, The synthetic data, generated using pysnptools do not involve real patient information and therefore require no additional ethical approval.

Note that full information on the approval of the study protocol must also be provided in the manuscript.

Field-specific reporting

Please select the one below that is the best fit for your research. If you are not sure, read the appropriate sections before making your selection.

☒ Life sciences ☐ Behavioural & social sciences ☐ Ecological, evolutionary & environmental sciences

For a reference copy of the document with all sections, see nature.com/documents/nr-reporting-summary-flat.pdf

Life sciences study design

All studies must disclose on these points even when the disclosure is negative.

Sample size	Real-world data: We used the full datasets provided by dbGaP (bladder cancer dataset: n=13,060; AMD dataset: n=22,683) without any further size-based selection. Synthetic data: We generated synthetic datasets matching the sample sizes used in the SF-GWAS [1] experiments to allow direct comparisons between PP-GWAS and SF-GWAS. No sample size calculation was performed and we used the same ones reported in SF-GWAS.<br><br>[1] Cho, Hyunghoon, et al. "Secure and federated genome-wide association studies for biobank-scale datasets." Nature Genetics (2025): 1-6.
Data exclusions	No data were excluded from any of the analyses.
Replication	We have made our code publicly available on GitHub, along with a Conda environment file and a pip requirements file (YML and TXT formats, respectively). To ensure reproducibility, we replicated the experiments across multiple computing environments: a local machine, Google Cloud, a local high-performance computing cluster, and the de.NBI Cloud cluster. In all cases, the code produced consistent results.
Randomization	Randomization was not applicable to this study, as we performed retrospective analyses of existing datasets rather than a prospective randomized experiment.
Blinding	Blinding was not relevant to our design. The datasets from dbGaP were de-identified, and our synthetic data contained no identifying information.

Reporting for specific materials, systems and methods

We require information from authors about some types of materials, experimental systems and methods used in many studies. Here, indicate whether each material, system or method listed is relevant to your study. If you are not sure if a list item applies to your research, read the appropriate section before selecting a response.

Materials & experimental systems

n/a	Involved in the study	
☒	☐	Antibodies
☒	☐	Eukaryotic cell lines
☒	☐	Palaeontology and archaeology
☒	☐	Animals and other organisms
☐	☒	Clinical data
☒	☐	Dual use research of concern
☒	☐	Plants

Methods

n/a	Involved in the study	
☒	☐	ChIP-seq
☒	☐	Flow cytometry
☒	☐	MRI-based neuroimaging

Clinical data

Policy information about clinical studies

All manuscripts should comply with the ICMJE guidelines for publication of clinical research and a completed CONSORT checklist must be included with all submissions.

Clinical trial registration	This study did not involve a prospective clinical trial. We conducted a secondary analysis of existing data from dbGaP.
Study protocol	The original data were collected under protocols approved by the respective Institutional Review Boards (IRBs). Full details of those protocols can be found in the dbGaP documentation for each dataset (accession numbers phs000346.v2.p2 and phs001039.v1.p1). We did not create a separate protocol for this secondary analysis.
Data collection	All data were collected previously by the original study investigators and made available through dbGaP. The bladder cancer dataset (n=13,060) and the age-related macular degeneration (AMD) dataset (n=22,683) were provided in de-identified form. No new data collection was performed for this study. Further information on the settings, time period, and recruitment can be found in the respective dbGaP records.
Outcomes	Our analysis focused on evaluating the performance (speed and scalability) and accuracy (p-value comparisons) of PP-GWAS against alternative GWAS methods. No new clinical outcomes were defined or measured in this secondary analysis.

Plants

Seed stocks	*Report on the source of all seed stocks or other plant material used. If applicable, state the seed stock centre and catalogue number. If plant specimens were collected from the field, describe the collection location, date and sampling procedures.*
Novel plant genotypes	*Describe the methods by which all novel plant genotypes were produced. This includes those generated by transgenic approaches, gene editing, chemical/radiation-based mutagenesis and hybridization. For transgenic lines, describe the transformation method, the number of independent lines analyzed and the generation upon which experiments were performed. For gene-edited lines, describe the editor used, the endogenous sequence targeted for editing, the targeting guide RNA sequence (if applicable) and how the editor was applied.*
Authentication	*Describe any authentication procedures for each seed stock used or novel genotype generated. Describe any experiments used to assess the effect of a mutation and, where applicable, how potential secondary effects (e.g. second site T-DNA insertions, mosiacism, off-target gene editing) were examined.*
